# Overexpression of microRNA-301b accelerates hippocampal microglia activation and cognitive impairment in mice with depressive-like behavior through the NF-κB signaling pathway

**DOI:** 10.1038/s41419-019-1522-4

**Published:** 2019-04-08

**Authors:** Chao-Zhi Tang, Dong-Fang Zhang, Jun-Tang Yang, Qing-Hui Liu, Ya-Ru Wang, Wen-Sheng Wang

**Affiliations:** Laboratory of Molecular Medicine, College of Life Science, Henan Normal University, 453007 Xinxiang, People’s Republic of China

**Keywords:** Cell biology, Molecular biology

## Abstract

Depression is a condition with a complex etiological pattern, whose effective treatments are highly limited. MicroRNAs (miRNAs) have been investigated in intensive studies owing to their involvement in pathophysiology of mood disorders. The current study aimed to elucidate the role of miR-301b in hippocampus in mouse models of depressive-like behavior. Microarray-based prediction identified the differentially expressed gene neuronal pentraxin II (NPTX2) related to mental depression. Next, the putative miR-301b binding sites on the 3′UTR of NPTX2 were verified. Then the effect of miR-301b on cognitive function of mice with depressive-like behavior was analyzed using the Morris water maze test. In addition, the regulation of miR-301b to NPTX2 and activation of NF-κB signaling pathway was assessed. Following that, the microglia activation and inflammation in hippocampus were evaluated, with the expressions of inflammatory factors being examined. At last, microglia were flow cytometrically sorted and the inflammatory reaction was also assessed in vitro. The obtained findings revealed that miR-301b targeted and negatively regulated NPTX2. Moreover, overexpressed miR-301b activated the NF-κB signaling pathway, as reflected by increasing protein expressions of p-NF-κB. Upregulated miR-301b accelerated cognitive impairment in mice with depressive-like behavior. In addition, overexpression of miR-301b activated microglia and stimulated inflammation in hippocampus, accompanied by enhanced release of tumor necrosis factor-α (TNF-α), interleukin-Iβ (IL-Iβ) and cyclooxygenase-2(COX-2). Taken together, the evidence provided by the current study indicated that overexpression of miR-301b augmented hippocampal microglia activation, thus exacerbating cognitive impairment and inflammation in mice with depressive-like behavior by activating the NF-κB signaling pathway.

## Introduction

Depression is a common psychiatric disorder, affecting approximately 300 million people across the world. Epidemiological reports have predicted that depression could be the leading cause of disease burden by 2030^1^. Depression is a potentially life-threatening disease that is accompanied with symptoms of anhedonia, sleep and appetite disturbances, low mood, in addition to feelings of despair, shame, and guilt^2^. Depression not only causes alterations to the emotional state, but may also consequently lead to cognitive impairments^3^. The onset of depression is closely associated with unhealthy levels of stress, damaged neurogenesis and defects in synaptic plasticity^4^. Microglia function as the macrophages of the central nervous system. Hippocampal microglial activation has been reported to promote the release of inflammatory factors that result in the disruption of neuroplasticity, deteriorate cognitive function and ultimately lead to exacerbation of the condition of depression^5,6^. Although several psychopharmacological agents have been employed in the treatment of depression, antidepressant therapy often brings about ineffective results and sometimes aggravates depression among a subsect of vulnerable patients suffering from depression^7,8^. Currently, there is a void for therapeutics that can selectively target the pathophysiological factors involved with the onset of depression, without the side effects of the used antidepressants.

MicroRNAs (miRNAs), as key post-transcriptional regulators of gene expression, participate in the regulation of nearly every cellular process, and their aberrant expression is implicated in the pathogenesis and response to the treatment of depression^9^. Similarly, a previous report pointed out that miRNAs could serve as critical players in the pathophysiology of depression and potential biomarkers for the development of antidepressants^10^. The involvement of the miR-301 family has previously been uncovered in cell proliferation, migration, invasion, clonogenicity, microvessel density, and tumor growth^11^. Furthermore, miR-301a was previously reported to participate in the cell invasion of glioma through the activation of the Wnt/β-catenin pathway^12^. In addition, a recent study demonstrated that miR-301b positively regulates inflammatory responses by targeting c-Myb^13^. The initial microarray-based analysis predicted NPTX2 as a putative target of miR-301b. NPTX2, also commonly referred to as neuronal activity regulated protein (NARP), promotes excitatory synapse formation, in addition to learning and memory function^14^. Moreover, patients treated with antidepressants exhibit elevated levels of NPTX2 in the hippocampus^15^. Interestingly, the prognostic significance of the NPTX2-PTEN-NF-κB nexus in glioblastoma has been highlighted in a study of Shukla et al.^16^, which also suggested that NPTX2 inhibits NF-κB activity by reducing AKT via the p53-PTEN-mediated pathway. In addition, NF-κB activation has been demonstrated to exacerbate constant darkness-induced depression-like behavior, in addition to inhibiting hippocampal cell proliferation^17^. A previous study provided verification that miR-301b expression led to NF-κB activation in pancreatic carcinoma^11^. Therefore, the current study aims to investigate the effect of miR-301b on the activation of microglia in mouse models with depressive-like behaviors, in an attempt to shed new light on underlying mechanisms involved in cognitive impairment and inflammation.

## Results

### MiR-301b is predicted to affect depression by regulating NPTX2 and the NF-κB signaling pathway

**Fig. 1:**
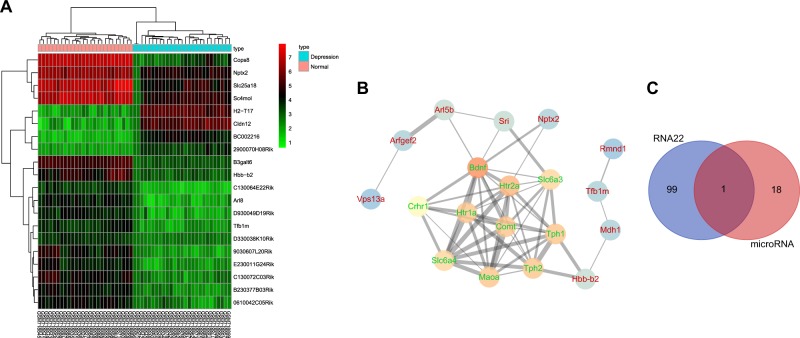
MiR-301b affects the progression of depression by regulating NPTX2 and the NF-κB signaling pathway. **a** The heat map of differential expression analysis in microarray dataset GSE29014. The *X*-axis represents the sample number, and the vertical axis represents the gene name. The upper dendrogram represents sample-type clustering. The upper color bars indicates the sample type. The left side of the dendrogram indicates gene expression level clustering. Each block shows the expression level of a gene in a sample, and different colors represent different expression levels. The upper right histogram indicates the color gradation with red indicating high expression and green indicating low expression. **b** Interaction network map of genes. Every circle in the graph represents a gene. The color of the circle indicates the core degree of the gene in the network as the higher the core degree is, the brighter the color is. The genes in green are the known genes for depression from database and the genes in red are significantly differentiated genes in microarray. The line between two circles indicates that there are direct or indirect interactions between the two genes. The thicker the lines, the higher the reliability of the interaction. **c** The predicted regulatory miRNAs of NPTX2. The blue circle indicates the first 100 miRNAs predicted in RNA22, and the right circle of represents the 18 miRNAs predicted in microRNA.org. The middle part represents the intersection of two database results, and the numbers in the panel indicates the number of miRNAs in each database

The GSE29014 expression profile was selected as the microarray data of genes associated with mental depression from the GEO database (https://www.ncbi.nlm.nih.gov/geo/). After differential expression analyses of brain tissue samples of mice with depressive-like behaviors and normal brain tissue samples, a total of 116 patients differentially expressed genes (DEGs) were obtained, among which 45 genes were significantly upregulated in the brain tissues of mice with depressive-like behaviors, and 71 genes were significantly downregulated in the brain tissues of mice with depressive-like behaviors. The top 20 genes with the largest fold-changes (differentially expressed) of 116 genes were selected to plot a heat map (Fig. 1a) and served for following gene association analysis. In the DisGeNET database, the genes associated with mental depression were retrieved, and the top 10 genes with the highest score were enrolled for subsequent analyses (Table 1). Correlation analysis between the first 20 significantly differentiated genes in GSE29014 and the 10 known genes was conducted using the STRING database and an interaction network map was plotted (Fig. 1b). The results showed that the 10 known genes were in the most central position. Among the DEGs, the NPTX2, Sri and hemoglobin Subunit Beta-b2 (Hbb-b2) genes were relatively more central, which exhibited evident interaction with the known genes of depression. There was a highly reliable interaction relationship between NPTX2 and brain derived neurotrophic factor (BDNF), which showed high scores in the known genes. Meanwhile, NPTX2 ranked first among DEGs between normal mouse brain tissues and brain tissues of mice with depressive-like behaviors by analyzing GSE29014. Further literature retrieval on the related signaling pathways of NPTX2 revealed that the NPTX2 gene can exert its function by regulating the NF-κB signaling pathway^18^, indicating close relation to depression^19–21^. Existing reports further suggested that the role of NPTX2 in depression may be achieved through the NF-κB signaling pathway. In order to further elucidate the functions of NPTX2 in depression, the RNA22 and microRNA.org databases were applied to predict the regulatory miRNAs of NPTX2 in mice. A total of 641 potential regulatory miRNAs were predicted from the RNA22 database, while only 19 miRNAs were obtained from the microRNA.org database. The top 100 miRNAs among 641 putative miRNAs which target NPTX2 were selected using the RNA22 database and a total of 19 putative miRNAs targeting NPTX2 were identified in the microRNA.org database. By performing the Venn diagram analysis, only miR-301b as the putative miRNA targeting NPTX2 was found to be intersected in the two databases (Fig. 1c). These findings demonstrated that miR-301b may affect the progression of depression by regulating NPTX2 and the NF-κB signaling pathway.

**Table 1:** The top 10 genes associated with mental depression, as identified by DisGeNET

Genes	Gene name	Score	PMIDs
*SLC6A4*	Solute carrier family 6 member 4	0.36	358
*BDNF*	Brain-derived neurotrophic factor	0.327	194
*HTR1A*	5-hydroxytryptamine receptor 1A	0.253	57
*HTR2A*	5-hydroxytryptamine receptor 2A	0.243	35
*COMT*	Catechol-*O*-methyltransferase	0.24	51
*MAOA*	Monoamine oxidase A	0.234	40
*TPH1*	Tryptophan hydroxylase 1	0.227	22
*TPH2*	Tryptophan hydroxylase 2	0.226	31
*CRHR1*	Corticotropin releasing hormone receptor 1	0.222	29
*SLC6A3*	Solute carrier family 6 member 3	0.222	17

Score of the reliability of the gene-disease pair, based on the type and number of sources where is reported, and the number of pmids; PMIDs: Total number of PMIDs supporting the association

### NPTX2 is verified as a target gene of miR-301b

**Fig. 2:**
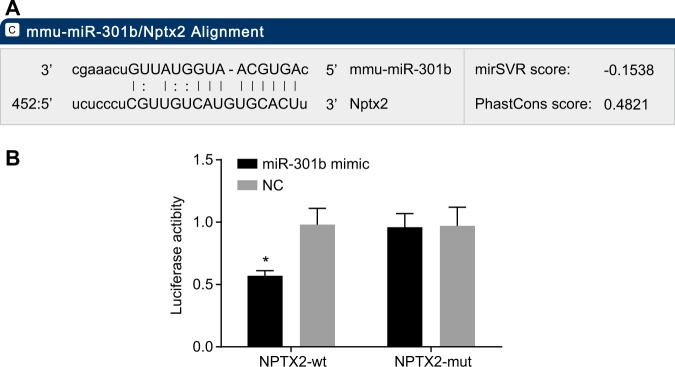
NPTX2 is determined as a target gene of miR-301b. **a** Predicted binding sites of miR-301b at NPTX2–3′UTR. **b** Comparison of luciferase activity in cells transfected with NPTX2-wt + miR-301b NC, NPTX2-wt + miR-301b mimics, NPTX2-mut + miR-301b NC, or NPTX2-mut + miR-301b mimics. Data are expressed by means ± standard deviation and analyzed by one-way ANOVA from three independent experiments. *p < 0.05, compared with the NPTX-wt + miR-301b NC group

A dual-luciferase reporter gene assay was applied to verification of 3′UTR of NPTX2 as recognized by miR-301b. According to the online tool (http://www.microrna.org) predictions, miRNA-301b was directly bound to the sequences of NPTX2-3′-UTR (Fig. 2a). The luciferase activity of the NPTX2-wt-3′-UTR in the miRNA-301b mimic group was found to be lower than that in the NC group (*p* < 0.05, Fig. 2b), while there were no differences in the luciferase activity of NPTX2-mut-3′-UTR between the NC and miR-301b mimic (*p* > 0.05) (Fig. 2b). Thus, NPTX2 was considered to be a potential target gene for miR-301b.

### Overexpression of miR-301b induces cognitive impairment

The effect of miR-301b on cognitive function in mice with depressive-like behavior was analyzed. Firstly, the Morris water maze method was employed to assess the cognitive impairment of the mice. Relative to the normal mice, mice with depressive-like behavior exhibited less resistance and longer static time in the tail suspension test, which suggested that the mice were in a desperate state. In addition, mice with depressive-like behavior displayed shorter escape latency and longer static time in the forced swimming test, which indicated that the mice were in a state of high tension. All in all, these findings indicated towards successful establishment of depressive-like behavior mice models.

**Fig. 3:**
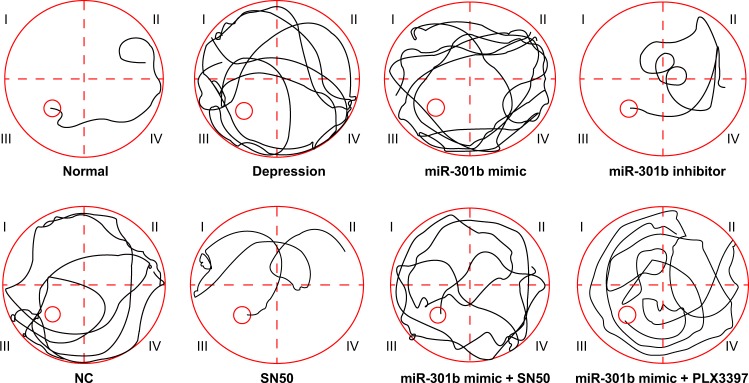
The trajectories of mice to find the platform in the place navigation test, which reflected the spatial learning and memory abilities of mice with depressive-like behaviors

In addition, the place navigation test (Fig. 3, Table 2) demonstrated that compared with the normal group, mice in other groups presented with increased finding platform times as well as escape latency (*p* < 0.05). Compared with the depression group, mice in the miR-301b mimic group showed increased time of finding the platform and escape latency (*p* < 0.05). Meanwhile, the mice in miR-301b inhibitor and SN50 groups showed decreased time of finding the platform and escape latency (*p* < 0.05). No significant differences were found in the time of finding the platform and escape latency in the NC, miR-301b mimic + SN50 and miR-301b mimic + PLX3397 group (*p* > 0.05). Compared with the miR-301b mimic group, mice in the miR-301b mimic + SN50 and miR-301b mimic + PLX3397 groups showed decreased time of finding the platform and escape latency (*p* < 0.05). Furthermore, the probe test (Table 2) illustrated that compared with the normal group, mice in other groups showed reduced residence time in the target area (*p* < 0.05). When compared with the depression group, mice in the miR-301b mimic group showed decreased residence time in the target area, while mice in miR-301b inhibitor, SN50, miR-301b mimic + SN50 and miR-301b mimic + PLX3397 groups showed heightened residence time in the target area (*p* < 0.05). No significant differences in the residence time were detected in the target area in the NC group (*p* > 0.05). Compared with the miR-301b mimic group, mice in the miR-301b mimic + SN50 and miR-301b mimic + PLX3397 groups exhibited significantly elevated residence time in the target area (*p* < 0.05). The aforementioned results indicated that overexpression of miR-301b accelerated the cognitive impairment in mice with depressive-like behavior, and the co-treatment of PLX3397 and SN50 could reverse the aggravation of cognitive impairment induced by overexpressed miR-301b.

**Table 2:** The place navigation test and the probe test in Morris water maze

	Escape latency (s)					Residence time (s)
Group	1d	2d	3d	4d	5d	6d
Normal	32.15 ± 1.15	27.45 ± 2.05	21.45 ± 1.85	15.35 ± 1.47	10.86 ± 2.88	59.27 ± 6.89
Depression	61.11 ± 2.15*	51.36 ± 1.17*	46.74 ± 1.27*	37.26 ± 2.01*	30.12 ± 1.46*	34.42 ± 4.82*
miR-301b mimic	71.97 ± 2.15*^#^	62.34 ± 2.25*^#^	55.19 ± 2.01*^#^	46.67 ± 2.11*^#^	39.54 ± 2.08*^#^	22.35 ± 2.40*^#^
miR-301b inhibitor	40.12 ± 1.68*^#&^	34.36 ± 1.55*^#&^	30.18 ± 2.88*^#&^	22.26 ± 2.97*^#&^	17.63 ± 1.48*^#&^	49.57 ± 5.58*^#&^
NC	60.58 ± 2.23*	49.67 ± 1.56*	45.44 ± 1.79*	35.62 ± 2.05*	27.88 ± 2.19*	33.94 ± 3.63*
SN50	45.81 ± 1.65*^#&^	36.54 ± 2.08*^#&^	30.20 ± 3.01*^#&^	23.40 ± 2.02*^#&^	17.56 ± 1.98*^#&^	46.56 ± 5.06*^#&^
miR-301b mimic + SN50	59.86 ± 2.47*^&^	50.37 ± 1.28*^&^	44.26 ± 1.34*^&^	36.17 ± 2.14*^&^	28.36 ± 1.75*^&^	35.47 ± 3.56*^&^
miR-301b mimic + PLX3397	62.34 ± 2.38*^&^	52.03 ± 1.35*^&^	47.23 ± 1.58*^&^	34.83 ± 2.21*^&^	29.47 ± 2.03*^&^	36.58 ± 3.24*^&^

Data are expressed by means ± standard deviation and analyzed by one-way ANOVA from three independent experiments

*p < 0.05 compared with the normal group

^#^*p *< 0.05 compared with the depression group

^&^*p *< 0.05 compared with the miR-301b mimic group

### Overexpression of miR-301b decreases the protein expression of NPTX2 while increasing that of p-NF-κB

**Fig. 4:**
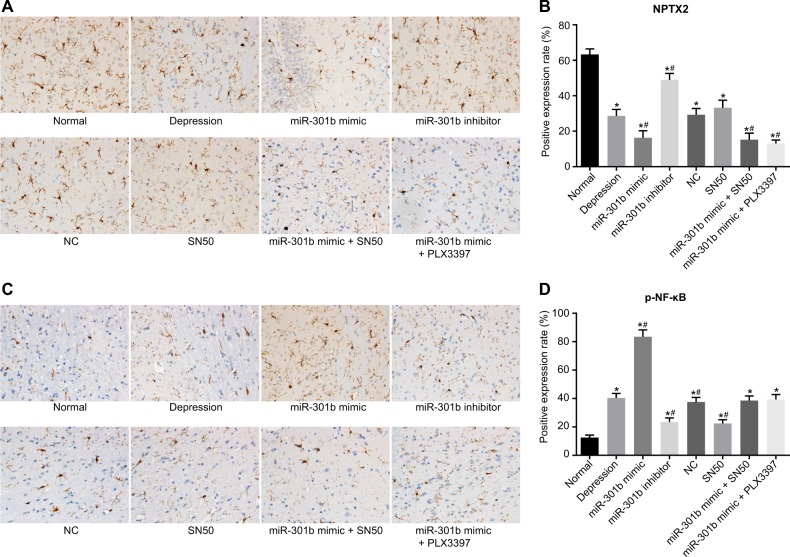
Overexpression of miR-301b could downregulate the protein expression of NPTX2 while upregulating that of p-NF-κB. **a** The immunohistochemical staining of NPTX2 protein in hippocampus of mice in each group (×400). **b** Quantitation of NPTX2 protein expressions in hippocampus of mice in each group. **c** The immunohistochemical staining of p-NF-κB protein expressions in hippocampus of mice in each group (×400). **d** Quantitation of p-NF-κB protein expressions in hippocampus of mice in each group. Data are expressed by means ± standard deviation and analyzed by one-way ANOVA from three independent experiments. *p < 0.05, compared with the normal group. ^#^*p* < 0.05, compared with the depression group

The protein expression levels of NPTX2 and p-NF-κB in brain tissues were observed and quantified following immunohistochemistry (Fig. 4), in which brown or brownish-yellow coloration represented positive expression. The NPTX2 protein expression was found to be reduced among the other groups in comparison with the normal group (*p* < 0.05). In contrast to the depression group, the miR-301b mimic, miR-301b mimic + SN50 and miR-301b mimic + PLX3397 group exhibited reduced protein expression of NPTX2, while the miR-301b inhibitor group showed elevated levels (*p* < 0.05). No distinct differences were detected in the NC and SN50 groups (*p* > 0.05). In contrast to the miR-301b mimic group, the miR-301b mimic + PLX3397 and miR-301b mimic + SN50 groups demonstrated no significant differences in the protein expression of NPTX2 (*p* > 0.05) (Fig. 4a, b).

In addition, the protein expression of p-NF-κB was observed using immunohistochemistry. As shown in Fig. 4c and d, p-NF-κB protein expression was represented by brownish-yellow particles localized to the cytoplasm (in triangle, fusiform and short-rod shapes) and was translocated to the nucleus. In contrast to the normal group, elevations in the protein expression of p-NF-κB were recorded among the other groups (*p* < 0.05). Compared to the depression group, the miR-301b inhibitor and SN50 groups displayed obviously reduced p-NF-κB protein expression while the miR-301b mimic group showed opposite trends (*p* < 0.05), and no significant differences were found in the NC, miR-301b mimic + SN50 and miR-301b mimic + PLX3397 groups (*p* > 0.05). In addition, no significant differences were observed in relation to p-NF-κB protein expression between the miR-301b inhibitor and SN50 groups (*p* > 0.05). These results indicated that overexpressed miR-301b could downregulate the protein expression of NPTX2 while leading to upregulation of the protein expression of p-NF-κB.

### Overexpression of miR-301b activates microglia in hippocampus

**Fig. 5:**
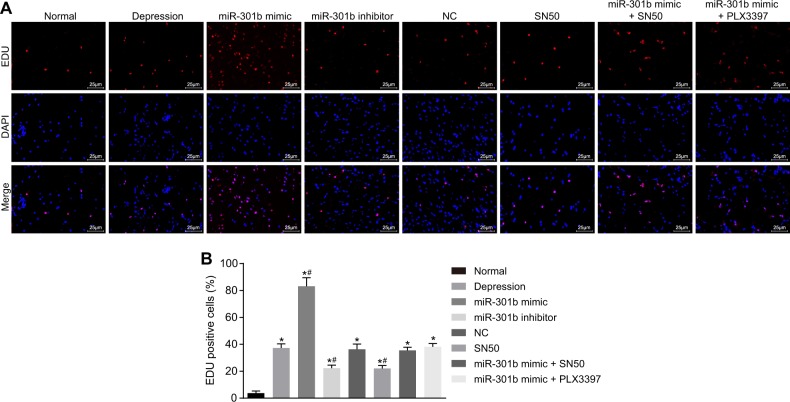
Overexpressed miR-301b activates microglia in the hippocampus of mice. **a** Iba1 immunofluorescence staining revealing the activation status of microglia in hippocampus of mice. **b** Quantitation of the number of Iba1-positive cells in hippocampus of mice in each group. Data are expressed by means ± standard deviation and analyzed by one-way ANOVA from three independent experiments. *p < 0.05, compared with the normal group. ^#^*p* < 0.05, compared with the depression group

The activation status of microglia was observed using Iba1 immunofluorescence staining (Fig. 5). Red staining indicated Iba1-positive microglia, and the blue staining was observed in the nucleus. In the normal group, the Iba1-positive microglia presented with lightly stained short and narrow outgrowth, and little positive expression was observed in the hippocampus and frontal lobe regions. Whereas, the other groups showed increased number of Iba1-positive cells, enlarged cell bodies, relatively more intense staining, and significantly increased number of microglia (*p* < 0.05). At the same time, versus the depression group, the number of Iba1-positive cells in the hippocampus and frontal lobe regions was found to be decreased, while the staining became lighter, and the number of microglia was decreased in the miR-301b inhibitor and SN50 groups. However, the miR-301b mimic group presented with opposite trends, as the number of Iba1-positive microglia was found to be increased (*p* < 0.05), indicating promoted microglia activation. In response to the co-treatment of miR-301b mimic + SN50 and miR-301b mimic + PLX3397, the number of microglia was not significantly different from the depression and NC groups (*p* > 0.05). These results indicate that reduced expression of miR-301b suppresses the activation of microglia in the hippocampus and frontal lobe regions of mice with depressive-like behavior.

### Overexpression of miR-301b induces inflammation in hippocampus

The expression of inflammatory factors, including tumor necrosis factor-α (TNF-α), interleukin-Iβ (IL-Iβ), and cyclooxygenase-2 (COX-2), in the hippocampus was examined by ELISA. As depicted in Table 3, compared with the normal group, the levels of TNF-α, IL-Iβ, and COX-2 were found to be enhanced in other groups (*p* < 0.05). The levels did not differ significantly between the depression group and the NC group (*p* > 0.05). Compared with the depression group, the miR-301b inhibitor and SN50 groups demonstrated reduced levels of TNF-α, IL-Iβ and COX-2 (*p* < 0.05), and opposite trends were observed in the miR-301b mimic group (*p* < 0.05). In addition, the levels of TNF-α, IL-Iβ and COX-2 were found to be not significantly different in the miR-301b mimic + SN50 and miR-301b mimic + PLX3397 groups, as compared with the depression and NC groups (*p* > 0.05). The results suggested that overexpression of miR-301b could stimulate the release of inflammatory factors in the hippocampus in mice with depressive-like behaviors, while SN50 and PLX3397 could diminish the increased inflammation induced by miR-301b.

**Table 3:** The expression of TNF-α, IL-Iβ, and COX-2 measured by ELISA

Groups	TNF-α (pg/mL)	IL-Iβ (pg/mL)	COX-2 (pg/mL)
Normal	20.11 ± 2.96	10.03 ± 3.65	6.95 ± 2.65
Depression	50.36 ± 3.25*	39.65 ± 2.66*	35.96 ± 1.96*
miR-301b mimic	82.32 ± 1.99*^#^	69.85 ± 2.65*^#^	58.96 ± 2.12*^#^
miR-301b inhibitor	35.65 ± 3.01*^#^	22.65 ± 2.99*^#^	21.23 ± 3.11*^#^
NC	52.65 ± 2.23*	36.99 ± 2.96*	39.56 ± 2.47*
SN50	38.68 ± 2.69*^#^	26.58 ± 2.58*^#^	23.25 ± 3.02*^#^
miR-301b mimic + SN50	51.23 ± 2.54*	37.34 ± 3.12*	36.22 ± 2.75*
miR-301b mimic + PLX3397	52.13 ± 3.24*	38.23 ± 3.25*	37.45 ± 2.87*

Data are expressed by means ± standard deviation and analyzed by one-way ANOVA from three independent experiments

*p < 0.05 compared with the normal group

^#^*p *< 0.05 compared with the depression group

### MiR-301b inhibits the transcriptional level of NPTX2

**Fig. 6:**
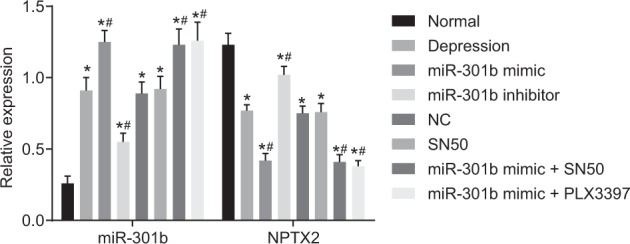
MiR-301b inhibits the transcriptional levels of NPTX2. Data are expressed by means ± standard deviation and analyzed by one-way ANOVA from three independent experiments. *p < 0.05, compared with the normal group. ^#^*p* < 0.05, compared with the depression group

MiR-301b expression and mRNA expression of NPTX2 were detected by RT-qPCR in order to further identify the regulation of miR-301b on NPTX2, with the results displayed in Fig. 6. Compared with the normal group, other groups demonstrated significantly promoted miR-301b expression while that of NPTX2 was diminished (*p* < 0.05). Relative to the depression group, the miR-301b mimic, miR-301b mimic + PLX3397 and miR-301b mimic + SN50 groups displayed enhanced expression of miR-301b. In addition, the miR-301b mimic and miR-301b mimic + PLX3397 groups showed reduced expression of NPTX2 (*p* < 0.05). In addition, the miR-301b inhibitor group showed markedly reduced expression of miR-301b, while that of NPTX2 was significantly elevated (*p* < 0.05). Besides, miR-301b and NPTX2 expression was not significantly different between the NC and SN50 groups (*p* > 0.05). No significant differences were found in miR-301b and NPTX2 expressions between the miR-301b mimic and miR-301b mimic + SN50 groups (*p* > 0.05). These results indicated that overexpression of miR-301b could downregulate the mRNA expression of NPTX2.

### Overexpression of miR-301b decreases the expression of NPTX2 while increasing that of p-NF-κB, TNF-α, IL-Iβ, and COX-2

**Fig. 7:**
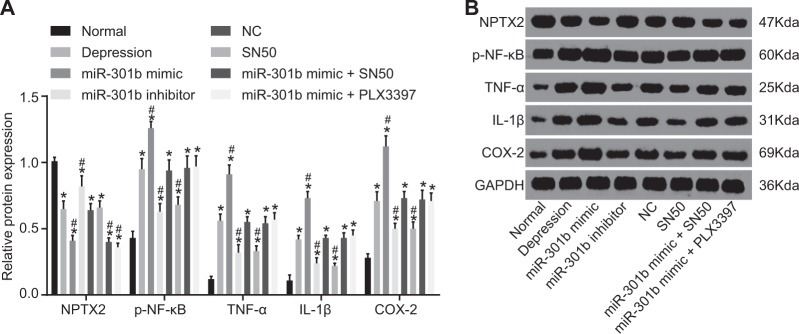
Overexpression of miR-301b reduces the protein expression of NPTX2 while elevating that of p-NF-κB, TNF-α, IL-Iβ, and COX-2. **a** Quantitation of the relative protein expressions of NPTX2, p-NF-κB, TNF-α, IL-Iβ, and COX-2 in each group. **b** The gray value of NPTX2, p-NF-κB, TNF-α, IL-Iβ, and COX-2 protein bands detected by western blot analysis in each group. Data are expressed by means ± standard deviation and analyzed by one-way ANOVA from three independent experiments. *p < 0.05, compared with the normal group. ^#^*p* < 0.05, compared with the depression group

Next, we performed western blot analysis in order to examine the protein expression of NPTX2, NF-κB, TNF-α, IL-Iβ, and COX-2, so as to confirm the findings of the above-mentioned PCR detection. The results (Fig. 7) indicated that compared with the normal group, the expression of NPTX2 was found to be decreased while that of p-NF-κB, TNF-α, IL-Iβ, and COX-2 was increased in other groups (*p* < 0.05). Relative to the depression group, the miR-301b mimic and miR-301b mimic + PLX3397 groups showed obviously decreased expressions of NPTX2, and the miR-301b mimic group displayed increased expressions of p-NF-κB, TNF-α, IL-Iβ, and COX-2 (*p* < 0.05). In addition, the treatment of miR-301b inhibitor led to increased expression of NPTX2 and decreased expression of p-NF-κB, TNF-α, IL-Iβ, and COX-2 (*p* < 0.05). NPTX2 expression in the SN50 group did not differ from the depression group (*p* > 0.05); however, the expression of p-NF-κB, TNF-α, IL-Iβ, and COX-2 was found to be decreased in the SN50 group (*p* < 0.05). The miR-301b mimic + SN50 and miR-301b mimic + PLX3397 groups displayed no significant differences in the expression of NPTX2, p-NF-κB, TNF-α, IL-Iβ, and COX-2 (*p* > 0.05). The aforementioned results indicated that overexpressed miR-301b promoted the expression of inflammatory factors by activating the NF-κB signaling pathway.

### Overexpression of miR-301b promotes inflammation in microglia in vitro

**Fig. 8:**
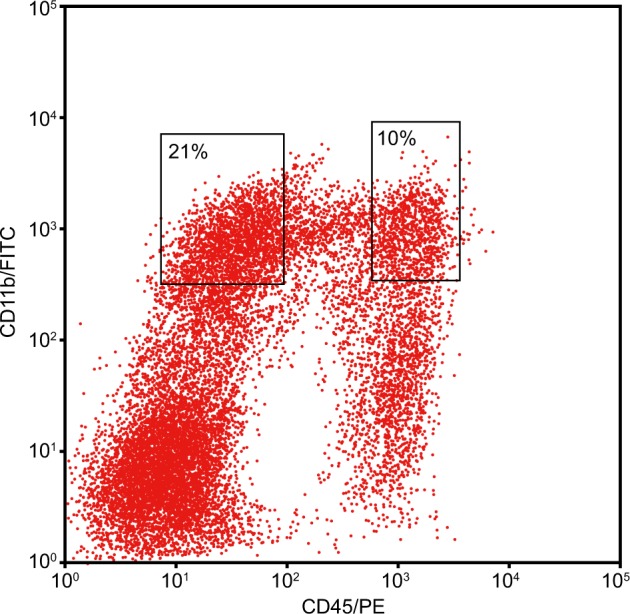
Flow cytometric characterization of microglia

Mononuclear cells obtained after trypsin digestion and Percoll density gradient centrifugation were labeled with antibody and flow cytometrically in order to obtain CD11b^+^ CD45^high^ and CD11b^+^ CD45^low^ microglia. The results showed that the ratio of activated microglia (CD11b^+^ CD45^high^) was 10%, and the ratio of quiescent microglia (CD11b^+^ CD45^low^) was 21% (Fig. 8). In addition, the levels of inflammatory factors (TNF-α, IL-Iβ, and COX-2) in supernatant were measured by ELISA (Table 4). Relative to the control group, the levels of TNF-α, IL-Iβ and COX-2 were found to be decreased in the miR-301b inhibitor and SN50 groups (*p* < 0.05), but increased in the miR-301b mimic group (*p* < 0.05). There were no differences detected in regard to the levels of inflammatory factors in the NC group and the miR-301b mimic + SN50 group, versus the control group (*p* > 0.05). In addition, no differences in relation to the levels of inflammatory factors were observed among the miR-301b inhibitor group and SN50 group (*p* > 0.05). The results of secretion of inflammatory factors of microglia in vitro were consistent with the results in vivo, suggesting that inflammatory factors were primarily produced by activated microglia.

**Table 4:** The levels of TNF-α, IL-Iβ, and COX-2 in supernatant by ELISA

Groups	TNF-α (pg/mL)	IL-Iβ (pg/mL)	COX-2 (pg/mL)
Control	41.32 ± 2.31	56.33 ± 3.65	11.96 ± 2.58
miR-301b mimic	62.36 ± 6.31*	81.36 ± 6.32*	22.29 ± 2.01*
miR-301b inhibitor	25.48 ± 2.03*	36.32 ± 3.18*	4.21 ± 2.36*
NC	44.12 ± 4.00	53.22 ± 4.12	15.63 ± 2.02
SN50	23.69 ± 2.11*	34.32 ± 3.36*	4.68 ± 2.31*
miR-301b mimic + SN50	42.35 ± 2.15	54.73 ± 3.14	13.26 ± 2.23

Data are expressed by means ± standard deviation and analyzed by one-way ANOVA from three independent experiments

*p < 0.05 compared with the control group

## Discussion

Depression is a debilitating psychiatric disease which drastically influences the quality of life of victims, affecting millions of people worldwide^22^. Interestingly, miRNAs have been proven to be effective regulators of protein expression, serving as potential regulators in pathways of brain development by mediating neurogenesis and synaptic plasticity^23^. In recent years, the role of miRNAs in the development of novel antidepressant treatments has been emphasized^24,25^. In the current study, we centered primarily upon the effect of miR-301b on depressive-like behaviors in mouse models and revealed that miR-301b could activate the microglia and facilitate the release of inflammatory factors such as TNF-α, IL-Iβ, and COX-2, thereby aggravating the cognitive impairment and inflammation in mice with depressive-like behaviors.

The current study demonstrated that the overexpression of miR-301b aggravated the damage to the spatial learning and memory abilities in mice with depressive-like behavior, reflecting enhanced cognitive impairment. Interestingly, when the NF-κB signaling pathway was inhibited or the microglia activation was blocked, the acceleration in cognitive impairment induced by overexpressed miR-301b could be reversed. Cognitive impairment, secondary to low mood, is a critical characteristic of depression, was previously reported to serve as a promising target for future interventions for depression^26^. In addition, microglia have been proven to have a role in neurogenesis, neuronal cell death, and synaptic interactions, and immune-response generating cytokines^4^. A previous study demonstrated that activated microglia aggravate the cognitive impairment in pilocarpine-induced status epilepticus^27^. Activation of microglia was further reported to be associated with depressive-like behavior^28^. MiRNA biogenesis is involved in various psychiatric disorders and the involvement may include stress response, neural plasticity and even the pathogenesis of depression^29^. Furthermore, the serum exosomal miR-301a expression was previously shown to be increased in glioma samples, and may be associated with accelerated pathological changes in glioma patients^30^. Similarly, another study reported that activation of miR-301a by the Wnt/β-catenin signaling pathway could promote glioma progression^12^. Moreover, β-catenin, as a key mediator of the Wnt signaling pathway, was previously demonstrated to contribute to the pathophysiology of mood disorders and hippocampal proliferation^31^. The knockout of NPTX2 may cause cognitive dysfunction in people diagnosed with Alzheimer’s Disease^32^. NPTX2 was intensely expressed in hippocampal sub-regions following treatment with antidepressants^15^.

The microarray-based analyses of the current study identified NPTX2 as a DEG associated with mice with depressive-like behaviors. In addition, the NPTX2 gene was found to exert effects by regulating the NF-κB signaling pathway. Notably, we further confirmed the existence of putative miR-301b binding sites on the 3′UTR of NPTX2. Owing to these findings, we deemed it is reasonable to hypothesize that miR-301b may affect the progression of depression by regulating NPTX2 and the NF-κB signaling pathway. NPTX2, also termed as NARP is involved in long-term neuronal plasticity^33^. Previous studies investigating the deletion of NPTX2 detected elevated activation of microglia in the dorsal horn of the spinal cord of mice functioning as inflammation regulators in the nervous system^34^. We also confirmed that miR-301b could inhibit NPTX2 at a transcriptional level. Furthermore, we demonstrated that overexpression of miR-301b decreased the protein expression of NPTX2 and activated the NF-κB signaling pathway.

Subsequently, we found that overexpression of miR-301b activated microglia and stimulated inflammation in hippocampus. Activation of the inflammatory system holds a significant role in the process of stress exposure, thus contributing to the risk of depression in populations^35^. In addition, Bibhabasu Hazra et al.^36^ demonstrated that miR-301b was abundantly increased in microglia during Japanese encephalitis virus infection. Recent data have revealed that psychiatric disorders could be regarded as inflammatory disorders, with microglial activation highlighted to be associated with an increase in the pro-inflammatory molecules of IL-Iβ and TNF-α^37^. The miR-301b family was previously demonstrated to negatively influence the treatment of the mental disorder of schizophrenia^38^ and stimulate pro-inflammatory factors interleukin (IL)-17A as well as TNF-α exacerbating intestinal mucosal inflammation^39^. Moreover, a prior study confirmed that miR-301b increased inflammation by repressing c-Myb^13^.

Furthermore, our findings displayed that the treatment of miR-301b mimic reduces the expression of NPTX2 and elevated that of p-NF-κB, TNF-α, IL-Iβ and COX-2. In addition, Shukla et al.^18^ suggested that NPTX2 inhibited the expression of NF-κB via suppression of AKT in the most widespread brain tumor, such as glioblastoma. Similarly, Funamizu et al.^11^ highlighted the role of miR-301b in promoting pancreatic carcinoma cell invasion via the NF-κB signaling pathway. Moreover, chrysin ameliorated neuro-inflammation by suppressing the release of pro-inflammatory factors TNF-α, IL-Iβ, and COX-2 and NF-κB signaling pathway in microglia cells^40^.

**Fig. 9:**
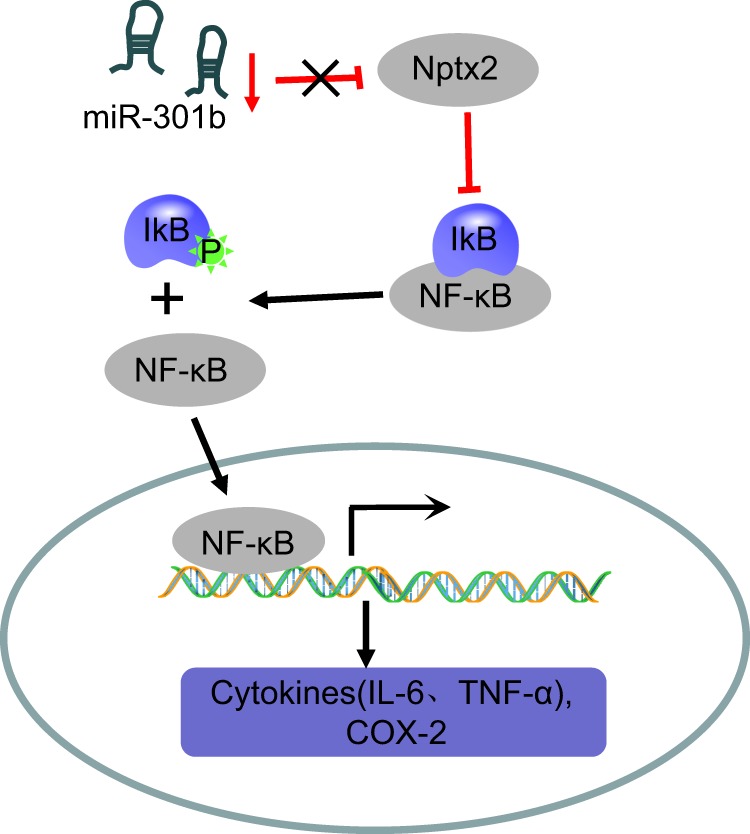
Schematic representation of the potential role of miR-301b as a contributor of depressive-like behaviors. MiR-301b activates the NF-κB signaling pathway through negative regulation of NPTX2, thus resulting in promoted inflammation, corresponding to increased levels of TNF-α, IL-Iβ, and COX-2

In conclusion, the current study delineated that miR-301b can act as a contributor to depression upon hippocampal microglial stimulation through the activation of the NF-κB signaling pathway by binding to NPTX2 (Fig. 9), thereby deteriorating cognitive impairment and inflammation in mouse models of depressive-like behavior. The key findings of our study may shed light for future studies investigating the binding of miR-301b to NPTX2 as therapeutic targets for the treatment of depression. Nevertheless, the influence of miR-301b on microglia activation in other regions of brain tissue was not investigated in the current study, which should be the subject of future experiments. Moreover, it would be of interest to further investigation the interaction between miR-301b and NPTX2 and the underlying mechanism by which activated microglia and inflammatory factors work to influence the incidence and development of depression.

## Methods and materials

### Ethical statement

All animal operations in the current study were performed in line with the local principles of management and use of experimental animals. The experiment procedure and animal operation protocols were pre-approved by the Institute of Laboratory Animal Science of Henan Normal University. All efforts were made in order to minimize both the number of animals used as well as their respective suffering.

### Microarray analysis

The GEO database (https://www.ncbi.nlm.nih.gov/geo/) was employed in order to retrieve the microarray data for genes associated with mental depression, and the GSE29014 expression profile was obtained, which comprised of 26 normal samples, 27 highly depressive samples and 25 lowly depressive samples. The “limma” package of the R language was used to analyze the difference between the normal samples and the highly depressive samples, with |logFC| > 2 and *p*-value < 0.05 set as the threshold. The 15 most significantly DEGs were selected to plot a heat map using the “pheatmap” package. In the DisGeNET database (http://www.disgenet.org/web/DisGeNET/menu), we searched the genes associated with mental depression, and top 10 genes with highest scores were selected for subsequent analysis. The relationship between the DEGs and the known genes was analyzed using the STRING database (https://string-db.org/), and the interaction network map was then plotted. Next, the RNA22 database (https://cm.jefferson.edu/rna22/Precomputed/) and microRNA.org database (http://34.236.212.39/microrna/home.do) were used to predict the miRNAs that regulate NPTX2 in mice. The intersection from the two aforementioned databases was analyzed through Venn Diagram (http://bioinformatics.psb.ugent.be/webtools/Venn/).

### Dual-luciferase reporter gene assay

The biological prediction website, microRNA.org, was employed in order to predict the target genes of miR-301b. Subsequently, a dual-luciferase reporter gene assay was conducted to verify whether NPTX2 was a direct target of miR-301b. NPTX2–3′-untranslated region (3′-UTR) wild type (wt) and mutant type (mut) were amplified and connected to psi-Cpsi-CHECK-2 vector (Promega Corp., Madison, Wisconsin, USA), and classified as NPTX2-wt and NPTX2-mut. HEK-293T cells (CRL-1415, Shanghai Xin Yu Biotech Co., Ltd, Shanghai, China) were co-transfected with 200 nmol/L miR-301b negative control (NC) or miR-301b mimic and 100 ng plasmids (NPTX2–3′-UTR-wt or NPTX2–3′-UTR-mut). After 48 h of transfection, the cells were then collected, lysed, and centrifuged for 3–5 min and the supernatant was subsequently collected. The firefly luciferase (LUC) assay kit (RG005, Shanghai Beyotime Biotechnology, Co., Ltd, Shanghai, China) was used to dissolve the renilla luciferase (RL) buffer, followed by firefly luciferase detection reagent respectively. Renilla detection working fluid was prepared by the addition of substrates and buffer (100 μL for each sample) at a proportion of 1:100. Each sample (50 μL) was mixed with 100 μL detection reagent using a gun. Cell lysate of the reporter gene served as a control. Next, 100 μL detection working fluid was added to the sample and mixed using a gun. After that, the relative luciferase units (RLU) were detected using fluorometer. The RL activities served as the internal control, and relative luciferase activity was determined using the ratio of RLU from firefly luciferase activity to RLU from RL activity^41^.

### Construction of expression vectors

MiR-301b NC/mimics/inhibitor shRNAs (Shanghai GenePharma Co., Ltd, Shanghai, China) were cloned into pSicoR plasmids, respectively. After digestion with Hpa I and Xho I and sequencing, the positive recombinant pSicoR-miR-301b expression vector, pCMV-VSV-G and pCMV-dR8.91 three plasmids (Biochemistry and Molecular biology Department of Medical Center of Fudan University) were co-transfected into HEK-293T cells (Shanghai Institute of Cell Biology, Shanghai, China) using liposome. After 48 h, the cells were centrifuged at 3000×*g* for 10 min at 4 °C, and the cell debris was removed with the supernatant collected. The supernatant was filtered using a 0.45 μM polyvinylidene fluoride (PVDF) membrane in order to obtain the lentiviral vector which was stored separately at −80 °C. HEK-293T cells (1 × 10^5^) were seeded into a 24-well plate, with 1 mL of Dulbecco minimum essential medium added (DMEM), cultured for 24 h at 37 °C with 5% CO_2_ in air, and infected. Virus (50 μL) was diluted with graded phosphate-buffered saline (PBS) (10^−^^1^–10^−6^) with each gradient set with 3 wells. The virus diluent (50 μL) was then added to the cells for infection purposes. After 24 h, fresh medium was added for further culture. After 48 h, the virus with available titer over 5 × 10^7^ TU mL^−1^ was regarded as the suitable vectors.

### Model establishment

The mouse models of depressive-like behavior were established by means of chronic unpredictable mild stress (CUMS) and separation. A total of 48 female Crlj:CD-1 (ICR) mice (4–6 weeks old, weighing 17–23 g) at specific-pathogen-free level were obtained from the Zhejiang Academy of Medical Science (Zhejiang, China. animal certification number was SCX K [Zhe] 2014–0001) and housed with temperature conditions between 25 and 28 °C. Mouse food was obtained from Tianjin Tian Yao Biotechnology Co., Ltd (Tianjin, China). The model establishment method was improved in accordance with the relevant documents^42,43^. Considerations were made in regard to the fact that the mice could potentially tolerate a single stressor at the same intensity, thus numerous unpredictable random ways were performed in order to alternately stimulate mice once a day for 21 days. The stressors included the following: fasting for 48 h, water deprivation for 24 h, tail clamping for 60 s, thermal stimulation at 45 °C for 5 min, over-hanging for 5 min, switching the day/night cycle, and cold stimulation at 4 °C for 5 min. All stressors were conducted three times within a period of 21 days, with each mouse housed in a separate cage at the same time.

### Assessment of depressive-like behavior in mice

The tail suspension test was conducted according as follows: the tails of mice were stuck to an iron beam using a medical proof fabric for a 6 min period of suspension. After 2 min, the accumulative static time of mice was assessed and compared to the other groups.

In addition, the forced swimming test was performed as follow: the mice were placed in a 1000 mL beaker with 25 °C warm water in order to allow their hind paws to touch the bottom of the water but fail to support the body weight. The mice were forced to swim for 6 min. After 2 min, the accumulative static time of the mice was assessed and compared to the other groups.

### Animal grouping

The mice were randomly assigned into the following groups: the normal group (ICR mice intravenously injected with normal saline), the depression group (mice with depressive-like behavior intravenously injected with normal saline), the NC group (mice with depressive-like behavior intravenously injected with miR-301b NC lentiviral vector), the miR-301b mimic group (mice with depressive-like behavior intravenously injected with lentiviral vector containing miR-301b mimic), the miR-301b inhibitor group (mice with depressive-like behavior intravenously injected with lentiviral vector containing miR-301b inhibitor), the SN50 group (mice with depressive-like behavior intravenously injected with SN50 reagent, SN50 is a cell osmotic peptide of nuclear localization sequence comprised of NF-κB p50 and could be linked to the hydrophobic region of K-FGF, thereby inhibiting the nuclear transposition of NF-κB), the miR-301b mimic + SN50 (mice with depressive-like behavior intravenously injected with lentiviral vector containing miR-301b mimic and SN50), and the miR-301b mimic + PLX3397 group (mice with depressive-like behavior intravenously injected with lentiviral vector containing miR-301b mimic and fed with food containing PLX3397. PLX3397 could deplete microglia in brain tissues), with 6 mice in each group. The administered drug dosage per group was set at 30 mg/kg. The normal and depression groups were injected with identical volumes of normal saline.

### Morris water maze method

After 3 days of drug administration, Ethovision XT (Noldus Information Technology, Wageningen, the Netherlands) was adopted in order to assess the behavior of the mice, and the place navigation test was performed to measure the learning ability of animals in the Morris water maze. In addition, the probe test was used to measure the memory retention in each mouse. The experiment lasted for a period of 6 days during which the first 5 days were dedicated to the place navigation test with the final day being dedicated for the probe test. The place navigation test was conducted as follows: the mice were permitted to swim freely for 2 min prior to the experiment, and then tested 4 times a day for a duration of 90 s each. The center of the 4 quadrants was randomly selected as the point of water entry and the mice were released into the water facing the pool wall, avoiding repetition of each point of water entry. The time spent finding and climbing the platform by the mice (5 s on the platform) was regarded as the escape latency. In the event that the mice failed to find the platform within 90 s, they would be gently guided by the experimenter to the platform, and the escape latency was recorded as 90 s. The mice were placed onto the platform for 20 s in order to block them from jumping into the water again. Mice were quickly dried and placed next to a heater after the completion of each experiment. Subsequently, the mean value of the escape latency of the 4 experiment sessions was calculated and recorded. Next, the probe test was performed as follows: On the 6th day of the experiment, the small platform under the water was removed, and the mice were placed in the water from the first quadrant and allowed to swim for 90 s. The residence time of crossing the quadrant of the platform was recorded in order to evaluate the memory retention ability of mice.

### Collection of brain tissues

After completion of the above experiments, the mice were anaesthetized via intraperitoneal injections with 2% pentobarbital sodium (0.005 mL/g). After fixing the limbs, a small incision was made in the middle of the chest to cut the ribs and separate the diaphragm. Next, the chest was opened and the heart was exposed. The aorta was pierced from the apex of heart, and the auricula dextra was cut to perfuse 100 mL of 0.9% normal saline into the heart. After cervical dislocation, the brain tissues were subsequently collected and divided into two halves. One half was fixed with 4% paraformaldehyde and embedded in paraffin after 12 h for immunohistochemistry, while the remaining half was treated with the separation of the hippocampus and cortex, and stored in liquid nitrogen at −80 °C for reverse transcription-quantitative polymerase chain reaction (RT-qPCR) and Western blot analysis.

### Immunohistochemistry

Tissue samples were slices into sections, dewaxed with xylene and dehydrated using gradient alcohol. The sections were added with 100 μL of Proteinase K (0.2 mg/mL) for antigen retrieval, placed at room temperature for 10 min and rinsed three times with 0.1 mol/L PBS (5 min per wash). The two-step method (PV-9000) was conducted to detect the protein levels of NPTX2. Antibody diluent was added to the NC group instead of the primary antibody, after which the sections were rinsed three times with 0.1 mol/L PBS (3 min per wash), incubated with 3% peroxidase at room temperature for 10 min. The sections were subsequently rinsed with PBS three times (3 min per wash), and then incubated with non-immune goat serum at room temperature for 30 min. After the supernatant was discarded, the sections were incubated with the primary antibody rabbit anti-mouse NPTX2 (ab69858, dilution ratio of 5 µg/mL, Abcam, Cambridge, UK), rabbit anti-mouse p-NF-κB (ab86299, dilution ratio of 1/500–1/2000, Abcam, Cambridge, UK) at 4 °C overnight. Following incubation, horseradish peroxidase (HRP)-labeled rabbit anti-rabbit secondary antibody (ab191866, dilution ratio of 0.02–0.2 µg/mL, Abcam, Cambridge, UK) was added to the sections for incubation at room temperature for 30 min. The sections were visualized with diaminobenzidine (DAB), counterstained with hematoxylin, and mounted on slides. Cells presenting with brown or brownish-yellow coloration in the cytoplasm were regarded as the positive cells. Next, 5 randomly selected fields of each section were observed under an optical microscope (×400). A total of 200 cells were observed in each field and the ratio of positive cells was calculated, and the mean value was obtained.

### Enzyme-linked immunosorbent assay (ELISA)

The hippocampus of the mice was separated on ice in accordance with the stereotactic anatomical atlas of mice with excess blood washed off by normal saline. After that, the hippocampus was placed into an eppendorf (EP) tube, sliced into pieces using ophthalmic scissors, and homogenized with a homogenizer. An antibody-coated 96-well plate was added with standard protein and samples (diluted in multiple proportion) in accordance with the instructions of ELISA Kit of TNF-α, IL-Iβ, and COX-2 (Boster Biological Technology Co., Ltd, Wuhan, Hubei Province, China), and shaken overnight at 4 °C. Next, the samples were rinsed using the cleaning solution attached with the kit and incubated with the primary antibody at 37 °C for 2 h. After washing, the HRP-labeled secondary antibody was added to the samples, and the samples were washed, and developed by trimethylbenzene (TMB). Optical density (OP) was measured using a 96-well microplate reader.

### Immunofluorescence

A set of frozen hippocampus sections (20 pm) were fixed in 4% paraformaldehyde and rinsed 3 times with 0.01 mol/L PBS 3 (10 min each). After being allowed to react with 0.3% H_2_O_2_ for 15 min, the sections were rinsed 3 times by 0.01 mol/L PBS (10 min each), and incubated in 0.3% Tritonx-100 solution for 30 min. The sections were then incubated with the primary antibody rabbit anti-mouse Iba1 (ab178847, dilution ratio of 1/100, Abcam, Cambridge, UK) at 37 °C for 2 h and then placed at 4 °C for 16–24 h. Subsequently, after three rinses with 0.01 mol/L PBS (10 min each), the sections were incubated with the HRP-labeled fluorescent rabbit anti-rabbit secondary antibody (ab191866, dilution ratio of 0.02–0.2 µg/mL, Abcam, Cambridge, UK) at 37 °C for 2 h or placed at room temperature for 2–4 h, followed by three rinses with 0.01 mol/L PBS three times (10 min each). After being mounted with 50% glycerol, the sections were observed and photographed under a fluorescence microscope. Each group was set with NC. NC sections were treated with same steps with the exception of the primary antibody addition, and positive control was not set.

### RT-qPCR

The brain tissues previously frozen in liquid nitrogen underwent a grinding process and total RNA content was extracted in accordance with the instructions of miRNeasy Mini Kit (Qiagen, Duesseldorf, Germany). A total of 5 μL RNA was diluted 20 times with ultra-pure RNase-free water. Next, ultraviolet spectrophotometer was applied to detect the OD values at 260 nm and 280 nm and the purity and concentration of RNA were calculated. The ratio of OD260/OD280 between 1.7 and 2.1 was considered to be of high purity. Subsequently, RNA was reversely transcribed into cDNA using a PCR amplifier. RT-qPCR was conducted with the ABI7500 quantitative PCR apparatus (7500, Applied Biosystems, Carlsbad, CA, USA) The reaction conditions were as follows: pre-denaturation at 95 °C for 10 min, 40 cycles of denaturation at 95 °C for 10 s, annealing at 60 °C for 20 s, and extension at 72 °C for 34 s respectively. Each pair of primers was set with three duplicated wells. The reaction system comprised of the following: 25 µL of VerQuest SYBR Green One-Step qRT-PCR Master Mix (2×), 0.5 µL of VeriQuest 100X RT Enzyme Mix for SYBR Green Assay, 2.5 µL of Forward Primer (10 µM), 2.5 µL of Reverse Primer (10 µM), 1 µL of Template RNA, and volume was up to 50 µL by RNase-free water and diethyl pyrocarbonate (DEPC)-treated water. Glyceraldehyde-3-phosphate dehydrogenase (GAPDH) and U6 served as the internal references. All aforementioned primers were synthesized by Beijing Genomics Institute (Beijing, China) (Table 5), and a solubility curve was used to assess the reliability of the obtained PCR results. The CT value (the threshold of the amplification curve) was calculated using the following formula: ΔCt = CT_(target gene)_ − CT_(internal reference)_. Relative quantitative method was adopted for calculation, and the relative expression of target gene was calculated according to 2^−^^ΔCt^ method^44^. Each experiment was repeated three times to obtain the mean value.

**Table 5:** Primer sequences for RT-qPCR

Targeted gene	Forward primer (5′–3′)	Reverse primer (5′–3′)
*miR-301b*	TGCGGGTGCTCTGACTAGG	TGGTCCCAGATGCTTTGACAAT
*NPTX2*	CTCAAGGACCGCTTGGAGAG	GGTCTCATTATGAAGCAGGGAC
*U6*	ACCCTGAGAAATACCCTCACAT	GACGACTGAGCCCCTGATG
*GAPDH*	AGGTCGGTGTGAACGGATTTG	GGGGTCGTTGATGGCAACA

*miR-301b* microRNA-301b, *NPTX2* neuronal pentraxin II, *GAPDH* glyceraldehyde-3-phosphate dehydrogenase, *RT-qPCR* reverse transcription-quantitative polymerase chain reaction

### Western blot analysis

The brain tissues previously frozen in liquid nitrogen were ground and lysed in RIPA lysis (Beyotime Biotechnology Co., Ltd, Shanghai, China), and then rinsed three times with pre-cooled PBS. After the cell samples were scraped down, they were transferred to a 1.5 mL centrifuge tube and pushed back and forth five times with a needle in order to allow the cells to completely lyse. The cells were centrifuged at 14,000×*g* for 10 min, and the supernatant was collected and preserved at −20 °C. The protein concentration was detected according to the instructions of bicinchoninic acid (BCA) kit (MultiSciences [Lianke] Biotech, Co., Ltd, Hangzhou, Zhejiang, China). The loading buffer was added to the extracted protein and boiled at 95 °C for 10 min. Next, the proteins were separated by means of polyacrylamide gel electrophoresis (the concentration of gel was prepared according to the molecular weight of the extracted protein) and transferred onto nitrocellulose membranes using the wet-spinning method. The membranes were blocked with 5% bovine serum albumin (BSA) at room temperature for 1 h, and then incubated with the primary antibodies, namely rabbit anti-mouse NPTX2 (ab69858, dilution ratio of 0.5–1 μg/mL), rabbit anti-mouse TNF-α (ab6671, dilution ratio of 1/500–1/2000), rabbit anti-mouse IL-Iβ (ab200478, dilution ratio of 2–3 µg/mL), rabbit anti-mouse COX-2 (ab15191), rabbit anti-mouse p-NF-κB (ab86299, dilution ratio of 1/2000–1/10,000) and rabbit anti-mouse GAPDH (ab22555, dilution ratio of 1/1000–1/5000), at 4 °C overnight. All aforementioned antibodies were purchased from Abcam (Cambridge, UK). After incubation, the membrane was washed and shaken with 1 × Tris-buffered saline with Tween 20 (TBST) at room temperature three times, for 5 min each. Next, the membrane was incubated with immunoglobulin G (IgG) rabbit anti-rabbit secondary antibodies (ab191866, Abcam, Cambridge, UK) at room temperature for 2 h, and rewashed three times with TBST (20 min each). The membranes were developed using the enhanced chemiluminescence (ECL) reagent and imaged using SmartView Pro 2000 (UVCI-2100, Major Science, Saratoga, California, USA). The gray values of the target band were analyzed using Quantity One software.

### Microglia cultivation in vitro

After the brain tissues in the hippocampal region of mice were digested by trypsin (Difco Laboratories, Detroit, Michigan, USA), mononuclear cells were isolated using density gradient centrifugation. Cell sediments were then collected and resuspended with the addition of 200–500 μL fluorescence activated cell sorting (FACS) buffer. After addition with FC block, the cells were placed on ice for 30 min for antigen blocking purposes. The cells were subsequently resuspended in 50 μL FACS buffer after centrifugation, and each of 10^6^ cells were added with 1 μL anti-CD11b-FITC and anti-CD45-PE (BD Biosciences, San Jose, CA, USA). After incubation on ice for 30 min in conditions void of light, the cells were centrifuged with the supernatant discarded, and resuspended with 400 μL FACS buffer. In addition, flow cytometry was conducted in order to sort the microglia. The cells were randomly classified into the following groups: the control group (minimum essential medium [MEM]), the NC group (MEM + miR-301b NC lentiviral vector), the miR-301b mimic group (MEM + miR-301b mimic lentiviral vector), the miR-301b inhibitor group (MEM + miR-301b inhibitor lentiviral vector), the SN50 group (MEM + SN50 reagent), and the miR-301b mimic + SN50 group (MEM + SN50 inhibitor). After 12 h, the levels of TNF-α, IL-Iβ, and COX-2 were evaluated by ELISA.

### Statistical analysis

Statistical analyses were performed using the statistical package for the social sciences (SPSS) version 21.0 (IBM, Armonk, NY, USA). All data were assessed for normality and homogeneity of variance. Measurement data were displayed as mean ± standard deviation. The *t*-tests were used for comparisons between two groups and one-way analysis of variance (ANOVA) was used for comparisons among multiple groups. In the event that the recorded data were normally distributed and exhibited homogeneous variance, comparisons between two groups were analyzed using the LSD test (SNK or *q* test); otherwise, comparisons between two groups were analyzed by the Tamhane’T2 test. A value of *p* < 0.05 was considered to be statistically significant.
